# Clinical characteristics and outcomes in patients with Takayasu arteritis coexisting with myocardial ischemia and neurological symptoms: A multicenter, long-term, follow-up study

**DOI:** 10.3389/fcvm.2022.948124

**Published:** 2022-08-03

**Authors:** Junting Huo, Bin Wang, LiJun Yu, Dewei Gao, Rui Cheng, Jiawei Wang, Xianliang Zhou, Tao Tian, Linggen Gao

**Affiliations:** ^1^Department of Neurology, Affiliated Chuiyangliu Hospital of Tsinghua University, Beijing, China; ^2^Department of Comprehensive Surgery, General Hospital of Chinese People’s Liberation Army and National Clinical Research Center for Geriatric Disease, Beijing, China; ^3^Department of Neurology, Beijing Tongren Hospital of Capital Medical University, Beijing, China; ^4^Department of Cardiology, FuWai Hospital and Cardiovascular Institute, Chinese Academy of Medical Sciences and Peking Union Medical College, Beijing, China

**Keywords:** Takayasu arteritis, myocardial ischemia, neurological symptoms, management strategy, prognosis

## Abstract

**Background:**

The incidence of coexisting myocardial ischemia and neurological symptoms in Takayasu arteritis (TA) is currently unknown. There is no standardized treatment algorithm in complex cases involving the coronary and intracranial arteries.

**Objective:**

This study aimed to describe the clinical characteristics and outcomes in patients with TA coexisting with myocardial ischemia and neurological symptoms.

**Methods:**

We retrospectively collected and assessed 1,580 patients with TA, and enrolled patients with myocardial ischemia and neurological symptoms from January 2002 to December 2021 in several hospitals. The incidence, clinical features, management strategy, and prognosis of these patients were evaluated.

**Results:**

Ninety-four (5.9%, 94/1,580) patients with TA coexisting with myocardial ischemia and neurological symptoms were included in the present study. Imaging results showed that the subclavian arteries were the most frequently affected arteries and 37 patients had intracranial vascular abnormalities, comprising the basilar artery (6.1%, 17/279), middle cerebral artery (2.5%, 7/279), anterior cerebral artery (2.9%, 8/279), and posterior cerebral artery (1.9%, 5/279). Among patients with neurological symptoms, 25 patients underwent percutaneous transluminal angioplasty and 20 patients underwent stent implantation. The most common site of stenosis was the ostial and proximal segments of the coronary artery, with 142 lesions among 188 (75.5%) lesions. Thirty-eight patients adopted interventional therapy, 21 patients underwent surgical treatment, and the remaining 35 patients received conservative treatment. There were 20 (21.27%, 20/94) late deaths during a mean follow-up of 57.79 months. The mortality rate in the conservative treatment group was significantly higher than that in the interventional therapy and surgical treatment groups.

**Conclusion:**

Patients with TA involving both the coronary and intracranial vessels are not rare. Stenosis and occlusion lesions most frequently involve the ostia and proximal segment of the arteries. Severe vascular lesions should be revascularized as soon as possible. These patients should be supplemented with glucocorticoids, antiplatelet, nitrates, and statins.

## Introduction

Takayasu arteritis (TA) is a chronic inflammatory disease of the aorta and its main branches. TA mainly shows stenosis or occlusion of different arteries. The epidemiological characteristics of TA mostly occur in young women before the age of 40 years. The etiology of TA is unclear, but may be related to genetic factors, immune factors, and estrogen concentrations. The clinical symptoms of TA are diverse, with mild symptomatic and severe life-threatening complications. The most common clinical symptoms are refractory hypertension, fever, fatigue, headache, and dizziness. The clinical manifestations of TA vary with the location of the lesion. The occlusive disease may cause catastrophic complications, such as stroke and acute myocardial infarction (MI). Recent advances in imaging technology allow relatively early diagnosis and treatment of serious complications of TA, such as intracranial and coronary artery involvement.

Catastrophic complications, such as MI (1) and stroke, are not rare and occur throughout the progressive course of TA (2). Several studies have reported that the incidence of coronary artery disease in patients with TA is 10–30% (3–5). Coronary involvement is detected by coronary CT angiography in up to 53.2–54.6% of patients with TA (3, 6). Serious complications, such as stroke, are caused by TA involving intracranial vessels (7–10). Some studies have reported neurological manifestations of patients with TA (10–12).

Currently, the clinical characteristics and treatment of patients with TA coexisting with myocardial ischemia and neurological manifestations have not been recognized or delineated clearly, and their outcomes have not been clarified. Therefore, this study aimed to evaluate the symptoms, signs, routine auxiliary examinations, angiography, treatment, and long-term outcomes of patients with TA coexisting with myocardial ischemia and neurological manifestations.

## Materials and methods

### Study population

We conducted a retrospective study and reviewed the electronic medical record system of patients with TA who were admitted to the Affiliated Chuiyangliu Hospital of Tsinghua University, General Hospital of Chinese People’s Liberation Army, and Fuwai Hospital and Cardiovascular Institute from January 2002 to December 2021. All the patients underwent aortic angiography, and CT angiography or MR angiography. All of them fulfilled the 1990 American College of Rheumatology criteria (13). We enrolled patients with TA coexisting with myocardial ischemia and neurological symptoms who were diagnosed based on angiographic (invasive or non-invasive) imaging, including standard angiography, CT angiography, or MR angiography.

### Data extraction

Demographic data, such as age, age of onset, sex, symptoms at admission, risk factors for coronary heart disease and stroke (including hypertension, diabetes, hyperlipidemia, and smoking), clinical manifestations, laboratory findings (e.g., erythrocyte sedimentation rate (ESR) and C-reactive protein (CRP) concentrations), echocardiographic findings (left ventricular size and ejection fraction), cerebral angiographic findings, coronary angiographic findings, and treatment (medical, intervention, or surgical), were collected.

### Definitions and assessment of treatment

The diagnosis of TA was based on clinical and angiographic features, with the exclusion of other differential diagnoses. The criteria of the American College of Rheumatology from 1990 were used as the inclusion criteria for patients in the study. Patients would be included, if they met at least three of the following six criteria: (1) an age of onset ≤ 40 years; (2) intermittent claudication; (3) pulsation of the brachial artery was weakened; (4) the systolic pressure difference between the two upper limbs was > 20 mm Hg; (5) there was a vascular murmur in the connecting area between the subclavian artery and the aorta; and (6) abnormal arteriography, except for arteriosclerosis and other causes. Patients with TA were classified into four types in accordance with the following criteria by Lupi-Herrera et al. (14): type I, involving the aortic arch and brachiocephalic artery; type II, involving the thoracoabdominal aorta, especially the renal artery; type III, type I + type II; and type IV, involving the pulmonary artery.

Emaciation was defined as weight loss caused by disease or some factors, which was more than 10% lower than the standard weight. A transient ischemic attack was defined as a transient blood supply shortage in the carotid artery or vertebrobasilar system, which resulted in focal cerebral ischemia and led to sudden, transient, and reversible neurological dysfunction. The attack lasted for several minutes, and the patient usually completely recovered within 30 min. Stroke was defined as focal cerebral dysfunction relevant to an ischemic lesion on MRI (15). In-stent restenosis was defined as a binary event with renarrowing of ≥ 50% of the vessel diameter, which was detected using coronary angiography (16).

### Cerebrovascular lesions

Patients with visual impairment or blindness, dyskinesia, aphasia, disturbance of consciousness, a coma, and other clinical symptoms were examined for cerebral vascular lesions of TA by whole cerebral angiography. Angiography was performed to show the degree of stenosis of the internal and external cranial arteries, the distribution of lesions, and the collateral circulation.

In this study, the following vascular stenosis rates were used for calculation. The vascular stenosis rate = (Normal inner diameter − Narrowest diameter)/Normal inner diameter × 100%. The vascular stenosis rate was divided into the following four grades: normal, mild, moderate, and severe. Normal stenosis indicated that the patients had no stenosis. Mild stenosis indicated that the rate of vascular stenosis was < 50%. Moderate stenosis indicated that the rate of vascular stenosis was 50–69%. Severe stenosis indicated that the rate of vascular stenosis was > 69%.

### Coronary angiographic findings

Patients with TA underwent coronary angiography, if they had a MI, pectoris angina, and an abnormal ECG or echocardiography with segmental abnormal movement in the left ventricular wall. According to the site of stenosis, angiography was classified into ostial lesions, proximal lesions, mid-segment lesions, and distal lesions. Patients were classified in accordance with the classification of the Coronary Artery Disease Reporting and Data System by a score of zero for normal coronary arteries to 5 for patients with at least one occluded coronary artery. The scoring system was as follows: 0, no plaque; 1, 1–24% stenosis; 2, 25–49% stenosis; 3, 50–69% stenosis; 4A, 70–99% stenosis; 4B, left main stenosis or three-vessel obstructive disease; and 5, total occlusion (17). Coronary angiography was recommended in patients with re-examination after revascularization or recurrent symptoms.

### Treatment strategies

The treatment strategies of TA mainly included medication (combined use of immunosuppressants and glucocorticoids), interventional therapy, endarterectomy, and vascular bypass grafting. The specific treatment strategy was decided by the doctor according to the patient’s condition and willingness for treatment. Inflammatory activity was evaluated regularly. The active status of TA was categorized into the following criteria: (i) emerging symptoms and/or signs (i.e., fever, myalgia, or weight loss); (ii) an elevated CRP concentration (> 0.9 mg/dl) or ESR (> 21 mm/h); and (iii) an imaging examination showed new arterial lesions or deterioration of original lesions.

### Follow-up

The included patients were followed-up at 1, 6, and 12 months and every year. The ESR and CRP concentrations were measured, and coronary angiography and coronary CT were performed when clinical symptoms occurred, especially in patients with recurrent pectoris angina and neurological symptoms. Restenosis was defined as intracranial or coronary artery stenosis > 50%. The overall mortality and the causes of death were evaluated. Composite events were all-cause death, MI, and stroke.

### Statistical analysis

Continuous variables with a normal distribution are reported as the mean with *SD*, and continuous variables with a non-normal distribution are reported as the median (interquartile range). Categorical variables are reported as frequencies. The association of each covariate with the outcome was determined using the chi-squared test or Fisher’s exact test. The Kaplan–Meier method was used to obtain freedom from event curves during follow-up. Differences were considered statistically significant at *p* < 0.05. Statistical analysis was performed with SPSS software (version 19).

## Results

### Demographic data and clinical characteristics

**FIGURE 1:**
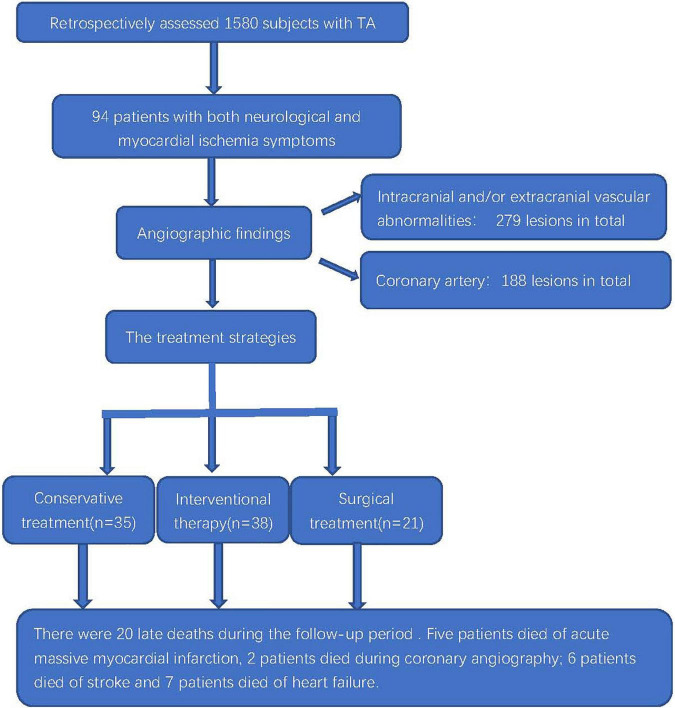
Flowchart of the clinical evaluation and outcomes of patients with TA coexisting with myocardial ischemia and neurological symptoms.

We reviewed the medical records of 1,580 inpatients with TA. There were 94 cases of TA with both the neurological and myocardial ischemia symptoms, and they accounted for 5.9% (94/1,580) of the hospitalized patients with TA in the same period (Figure 1). The baseline characteristics of the included patients are shown in Table 1. The mean age at symptom onset of the patients was 36.5 ± 12.3 years. The mean duration from symptom onset to the first hospitalization was 154.6 ± 81.7 months. The mean age of onset of cardiac ischemia or neurological symptoms was 38.8 ± 12.3 years. The female to male ratio was 5.7:1 (80/14).

**TABLE 1:** General clinical characteristics of patients with TA coexisting with myocardial ischemia and neurological symptoms.

General clinical characteristics	No. or value	Proportion (%)
Age at symptoms onset	25.9 ± 6.1	
Age of first onset of coronary ischemia	38.8 ± 8.5	
Female	80	85.1
**Inflammatory symptoms**		
Emaciation	16	17.0
Fever	9	9.6
Joint pains	10	10.6
Malaise	18	19.1
Carotid pathway pain	11	11.7
**Cardiovascular and cerebrovascular risk factors**		
Smoking	15	16.0
Diabetes mellitus	7	7.4
Hypertension	49	52.1
Hyperlipidemia	23	24.5
**Clinical covariates**		
Body mass index, kg/m^2^	23.4 ± 2.6	
Prednisone use	70	74.5
Glucocorticoid	94	100
Immunosuppressants	66	70.2
Statin use	27	28.7
Antiplatelet agents	80	85.1
ACEIs/ARBs	36	38.3
β-Blockers	30	31.9
**Laboratory values**		
C-reactive protein, mg/l	8.1 ± 3.8	
ESR, mm/h	15.6 ± 7.6	
Hemoglobin, g/dl	120.4 ± 18.9	
Lymphocyte,%	15.7 ± 9.0	
D-dimer, mg/ml	1.0 ± 0.6	
Creatine kinase, U/l	184.8 ± 200.9	
Cholesterol, mmol/L	4.2 ± 1.1	
LDL-C, mmol/L	2.5 ± 0.8	
Bilirubin, mmol/L	11.7 ± 7.0	
CK-MB, U/l	10.6 ± 6.0	
Myoglobin	59.9 ± 51.7	
Lactate dehydrogenase, U/l	158.8 ± 37.6	
Creatinine, μmol/L	77.5 ± 18.8	
Albumin, g/dl	28.9 ± 4.8	
Sodium, mmol/L	138.5 ± 4.1	
BNP, pg/ml	516.1 ± 858.4	
Disease activity, *n* (%)	29	30.9
**Classification of Takayasu arteritis**		
Type I	45	47.9
Type II	35	37.2
Type III	9	9.6
Type IV	5	5.3
**Treatment strategies**		
Conservative treatment	13	14.9
Interventional therapy	60	63.8
Surgical treatment	21	22.3
**Treatment strategies for CAL**		
Conservative treatment	35	37.2
PCA	10	10.6
PCI	28	29.7
Intervention only for CAL	13	13.8
Intervention for both CAL and I/R VA	25	26.6
Surgical treatment	21	22.3
**Treatment strategies for I/R VA**		
Conservative treatment	47	50.0
PTA	25	26.6
Stent implantation	20	21.3
Intravenous thrombolysis + stent implantation	2	2.1
Intervention only for I/R VA	22	23.4

TA, Takayasu arteritis; ACEIs/ARBs, angiotensin-converting enzyme inhibitors/angiotensin receptor blockers; ESR, erythrocyte sedimentation rate; LDL-C, low-density lipoprotein cholesterol; CK-MB, creatine kinase isoenzyme; BNP, brain natriuretic peptide; CAL, coronary artery lesions; PCA, percutaneous coronary angioplasty; PCI, percutaneous coronary stent implantation; I/R VA, intracranial and/or extracranial vascular abnormalities; PTA, percutaneous transluminal angioplasty.

Among the 94 patients, 64 (68.1%) patients had non-specific symptoms (Table 2), including 16 patients with emaciation, 9 patients with fever, 10 patients with joint pain, 18 patients with malaise, and 11 patients with carotid pathway pain. Sixty-eight (72.3%, 68/94) patients had a history of typical angina pectoris, 32 (34.0%, 32/94) patients had a MI, and 17 (18.1%, 17/94) patients had congestive heart failure. Twenty-six (27.7%, 26/94) patients had no typical history of chest pain. From the beginning of angina symptoms to the confirmation of coronary angiography, the course of the disease ranged from 1 month to 15 years. Neurological manifestations are shown in Table 3. Neurological symptoms comprised dizziness (80 cases, 85.1%), syncope (21 cases, 22.3%), headache (24 cases, 25.5%), visual impairment or blindness (23 cases, 24.5%), paresthesia (19 cases, 20.2%), dyskinesia (35 cases, 37.2%), aphasia (7 cases, 7.4%), and coma (2 cases, 2.1%). There were 23 cases of hyperlipidemia, 7 cases of diabetes, 15 cases of smoking, and 49 cases of hypertension.

**TABLE 2:** Cardiac symptoms and signs of patients with coronary artery lesions.

	No. or value	Proportion (%)
**Cardiac symptoms**		
Typical chest pain	68	72.3
Atypical, but still possible	26	27.7
Dyspnea	37	39.4
Palpitations	62	66.0
**ECG findings**		
ST-segment depression	73	77.7
ST-segment elevation	19	20.2
T-wave inversion	35	37.2
New pathological Q waves	7	7.4
LBBB	5	5.3
Any ischemic ECG changes	89	94.7
**Echocardiography**		
LVEF,%	59.6 ± 10.7	
Aortic regurgitation	9	9.6
Mitral regurgitation	13	13.8
Segmental wall motion abnormalities	21	22.3
Myocardial infarction	32	34.0
Congestive heart failure	17	18.1

ECG, electrocardiogram; AMI, acute myocardial infarction; LBBB, left bundle branch block; LVEF, left ventricular ejection fraction.

**TABLE 3:** Neurological manifestations of patients with TA.

Neurological manifestations	No.	Proportion (%)
Dizziness	80	85.1
Syncope	21	22.3
Headache	24	25.5
TIA	20	21.3
Visual impairment or blindness	23	24.5
Paresthesia	19	20.2
Dyskinesia	35	37.2
Aphasia	7	7.4
Disturbance of consciousness	9	9.6
Coma	2	2.1
Other clinical symptoms	34	36.2

TIA, transient ischemic attack.

According to the arteries involved, type I accounted for 47.9% (45/94) of cases, followed by type II in 35 (37.2%, 35/94), type III in 9 (9.6%, 9/94), and type IV in 5 (5.3%, 5/94).

**FIGURE 2:**
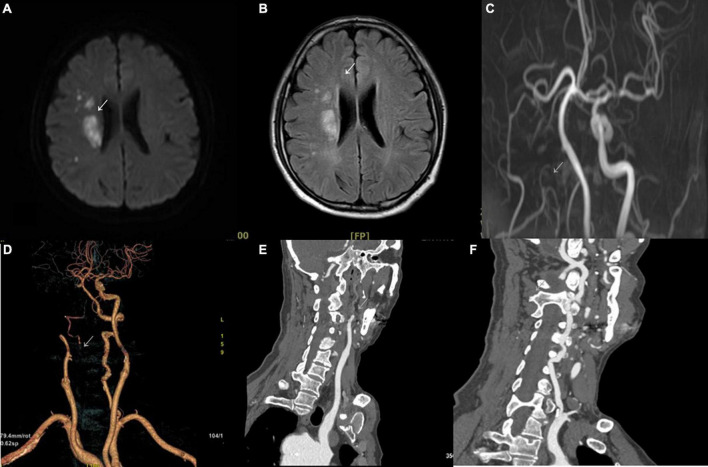
Brain MRI and CT angiography. **(A)** Diffusion-weighted imaging in the axial plane shows a high signal restriction in the right region beside the lateral ventricle (arrowhead) with a corresponding high signal in T2-weighted fluid-attenuated inversion recovery **(B)**. **(C)** Multicategorization recommendation adjusting shows occlusion of the right internal carotid artery (arrowhead). The arterial phase of CT angiography also shows occlusion in volume rendering imaging **(D)** and in the sagittal plane **(E)** (arrowhead). **(F)** Stenosis of the initial segment of the left vertebral artery can be seen (arrowhead).

**FIGURE 3:**
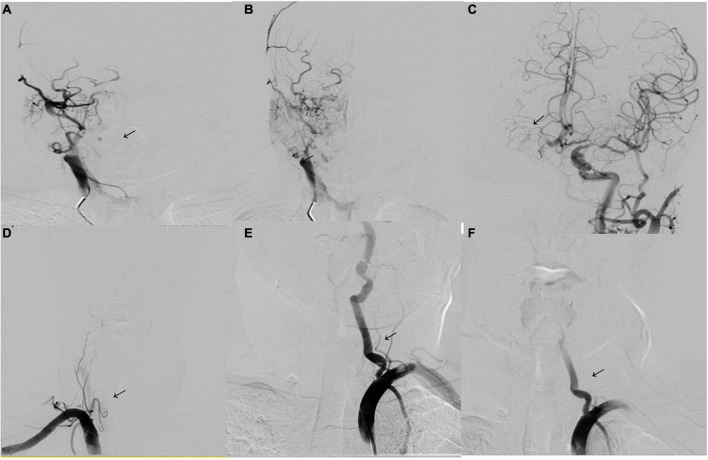
Digital subtraction angiography. **(A,B)** Digital subtraction angiography (DSA) shows occlusion of the right internal carotid artery (arrowhead). **(C)** DSA of the left internal carotid artery shows the formation of stenosis of the M1 segment of the left middle cerebral artery (arrowhead). The anterior communicating artery is open and supplies blood to the right internal carotid artery. **(D)** DSA of the right vertebral artery shows occlusion of the initial segment (arrowhead). **(E)** DSA of the left vertebral artery shows severe stenosis of the initial segment of the left vertebral artery (80%) (arrowhead). **(F)** Image showing placement of a vertebral artery stent (arrowhead).

**FIGURE 4:**
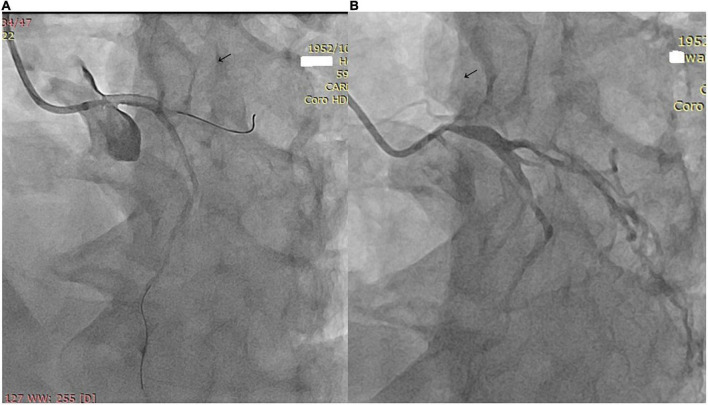
**(A)** Coronary angiography shows ostial stenosis of the left main coronary artery. **(B)** A 3.5 mm × 12 mm paclitaxel-eluting stent was implanted.

The imaging results of intracranial and/or extracranial vascular abnormalities and the detailed distribution of coronary involvement are shown in Tables 4, 5. Brain MRI, CT angiography (Figure 2), digital subtraction angiography (Figure 3), and coronary angiography (Figure 4) showed acute cerebral ischemia and ostial stenosis of the left main coronary artery. The imaging results showed that intracranial involvement was not rare. Subclavian lesions (31.2%, 87/279) were the most frequent, followed by common carotid lesions (27.2%, 76/279), internal carotid lesions (11.5%, 32/279), and external carotid lesions (10.4%, 29/279). Furthermore, cerebral angiography showed that 37 patients had intracranial vascular abnormalities, comprising the basilar artery (6.1%, 17/279), middle cerebral artery (2.5%, 7/279), anterior cerebral artery (2.9%, 8/279), and posterior cerebral artery (1.9%, 5/279).

**TABLE 4:** Imaging results of intracranial and/or extracranial vascular abnormalities of the patients.

Diseased vessels	Total lesions (*n*)	Stenosis 50–69%	Stenosis 70–99%	Occlusion (*n*)	Dilation	Aneurysm
Anterior cerebral artery	8	3	3	2	0	0
Middle cerebral artery	7	2	3	2	0	0
Posterior cerebral artery	5	2	2	1	0	0
Basilar artery	17	9	2	4	2	0
Internal carotid artery	32	8	4	15	5	0
External carotid artery	29	6	8	12	4	0
Common carotid artery	76	28	8	31	6	3
Vertebral arteries	18	5	4	7	2	0
Subclavian arteries	87	9	13	58	5	2
Total	279	72	47	132	24	5

**TABLE 5:** Imaging results of coronary artery lesions of the patients.

Variables	Total lesions (*n*)	Stenosis 50–70%	Stenosis 70–99%	Occlusion (*n*)	Dilation
**Diseased vessels**					
LM, *n*	43	23	17	0	3
LAD, *n*	57	32	17	4	4
LCX, *n*	39	17	15	7	0
RCA, *n*	49	24	16	5	4
**Location of coronary stenosis**					
Ostial coronary artery	56	29	22	4	1
Proximal segment	86	38	36	8	4
Middle segment	30	17	4	3	6
Distal segment	16	12	3	1	0

LM, left main coronary artery; LAD, left anterior descending artery; LCX, left circumflex; RCA, right coronary artery.

There were 188 coronary artery stenoses in 94 patients. The most common site of stenosis was the ostial and proximal segments of the coronary artery, with 142 (75.5%, 142/188) lesions. The ostial lesions of the opening of the coronary artery were serious, and there were 16 (8.5%, 16/188) occlusion lesions. The left anterior descending coronary artery was the most frequently involved vessel (30.3%, 57/188). The stenosis of 96 lesions was < 70%.

### Treatment strategies

All the patients were treated with glucocorticoids and/or immunosuppressive agents. Additionally, dual antiplatelet drugs (clopidogrel 75 mg and aspirin 100 mg daily) were suggested to be taken for 12 months after stent implantation. Prednisone was prescribed at a daily dose of 0.5 mg/kg/day for the first month, and then gradually tapered at a rate of 5 mg every 2 weeks down to 10 mg. Prednisone was decreased thereafter at a rate of 2.5 mg every 2 weeks to the minimum required dose, with a maintenance dosage of 5–10 mg/day for at least 1 year.

The treatment strategies were as follows. Thirteen patients adopted intervention only for coronary artery lesions (CALs), 22 patients adopted intervention only for intracranial and/or extracranial vascular abnormalities (I/R VAs), and 25 underwent intervention for both CAL and I/R VA. Twenty-one patients underwent surgical treatment such as bypass grafting, and the remaining 13 participants received conservative treatment.

The treatment of coronary lesions was as follows. Ten patients adopted interventional therapy of percutaneous coronary angioplasty and 28 patients adopted percutaneous coronary intervention (PCI). Twenty-one patients underwent surgical treatment such as bypass grafting, and the remaining 35 patients received conservative treatment. All the patients had detailed information about the implanted stents. The stents included 10 bare-metal stents and 30 drug-eluting stents. Additionally, 21 patients underwent coronary artery bypass grafting (CABG). Six patients used the left internal mammary artery, and 15 patients used saphenous venous grafts as grafts.

Among patients with intracranial and/or extracranial vascular abnormalities, 47 patients received conservative treatment, 25 patients underwent percutaneous transluminal angioplasty, and 20 patients underwent stent implantation. All the patients had detailed information about the implanted stents. The stents included 14 bare-metal stents and 8 drug-eluting stents. Two patients who presented with acute ischemic stroke were treated with intravenous thrombolysis and subsequent endovascular intervention.

Among 37 patients with renal artery abnormalities, 15 patients received conservative treatment, 10 patients underwent percutaneous transluminal angioplasty, and 12 patients underwent stent implantation. Among five patients with pulmonary artery abnormalities, two patients received conservative treatment, two patients underwent percutaneous transluminal angioplasty, and one patient underwent stent implantation.

Commonly used drugs after the operation included aspirin, clopidogrel, statins, β-receptor blockers, and angiotensin-converting enzyme inhibitors/angiotensin II receptor antagonists.

### Clinical outcomes

There were 20 (21.27%) late deaths during a mean follow-up of 133.6 months. During the follow-up, five patients died of acute massive MI, two patients died during coronary angiography (left main artery disease), six patients died of a stroke, and seven patients died of heart failure. Four patients developed bridge stenosis in the CABG group, and 12 patients developed in-stent restenosis in the PCI group. Among them, 13 patients were in the active status of TA. The multivariate Cox proportional regression analysis showed that the age of onset, active status, and hypertension were independently associated with composite events (Table 6).

**TABLE 6:** Risks related to main adverse events in patients with TA coexisting with myocardial ischemia and neurological symptoms.

Risk factors	Hazard ratio HR (95% CI)
Onset age	1.87 (1.40–2.71)
Active status	2.37 (1.77–2.95)
Hypertension	1.06 (1.02–1.46)
Hypercholesterolemia	1.04 (0.89–1.50)
Revascularization	0.52 (0.33–1.25)

**FIGURE 5:**
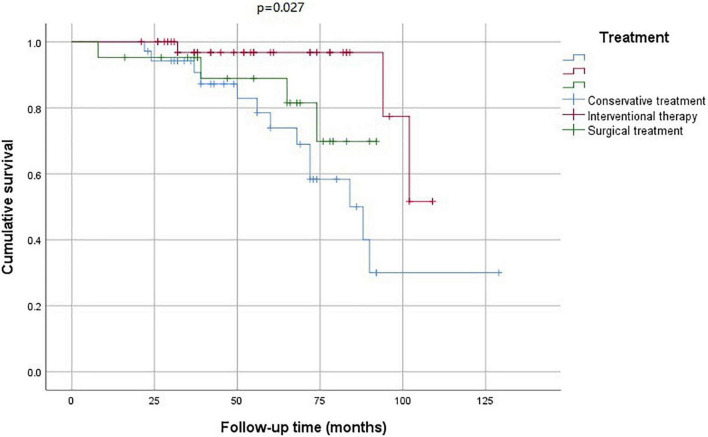
Survival analysis according to the treatment strategies.

**FIGURE 6:**
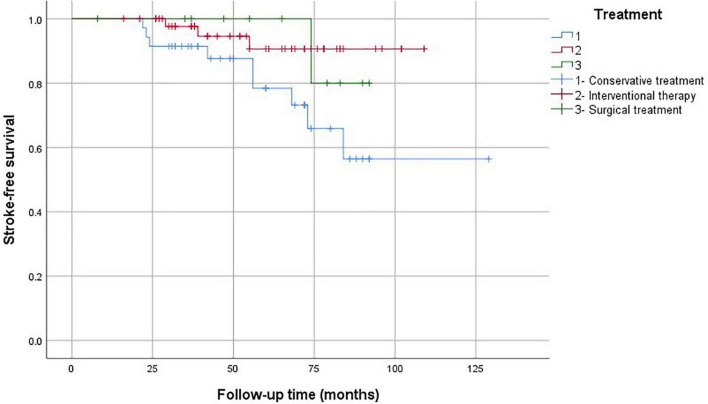
Stroke-free survival analysis according to the treatment strategies.

**FIGURE 7:**
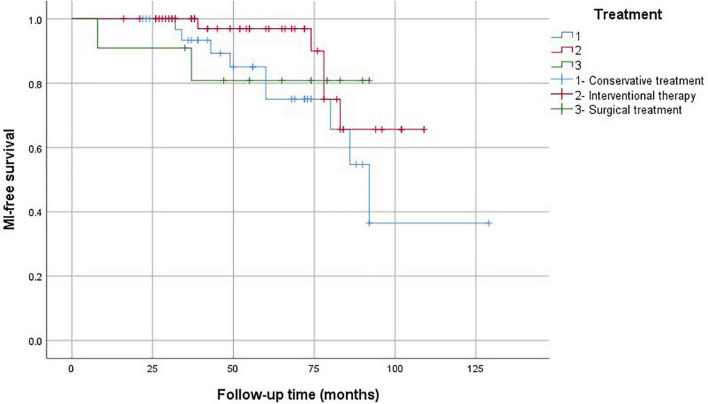
Myocardial infarction-free survival analysis according to the treatment strategies.

The Kaplan–Meier analysis showed that the mortality rate in the conservative treatment group was significantly higher than that in the interventional therapy and surgical treatment groups (Figure 5). The stroke-free survival and MI-free survival rates in the conservative treatment group were significantly lower than those in the interventional therapy and surgical treatment groups (Figures 6, 7).

## Discussion

In the present multicenter, retrospective study, we found that 5.9% of TA cases coexisting with myocardial ischemia and neurological symptoms usually occurred in young women and frequently involved the aorta and other branches. The mean age at onset of disease in our study was 25.9 years, which was in accordance with previous studies (12, 18). Multiple and severe stenotic or occlusive lesions in the coronary artery, superior arch artery, and intracranial artery may cause MI and stroke in patients with TA.

Coronary and cerebral angiographies are currently recognized as the gold standard for the diagnosis of cardiocerebral vascular stenosis. These examinations can clearly show the location, degree, scope, and collateral circulation of vascular lesions. All the included patients underwent coronary and cerebral angiographies. The angiographic results of this study suggest that TA often invades the opening of the trunk of the coronary artery or intracranial artery, but rarely involves the distal end. This study showed that, in young patients with peripheral artery disease (e.g., repeated chest tightness, chest pain, syncope, cerebrovascular events, and visual impairment), especially women, the possibility of TA involving the coronary artery, supra-arch vessels, and intracranial vessels should be considered. Additionally, coronary artery and intracranial angiography should be performed as early as possible to perform a clear diagnosis and early treatment.

MI and stroke are the most serious complications of TA, and they can cause serious cardiac insufficiency, malignant arrhythmia, neurological defects, and even life-threatening. Therefore, TA involves the coronary artery and intracranial vessels at the same time, and the prognosis is poor, which is a concern.

In this study, the mortality rate in the conservative treatment group was as high as 37.1%. We found that the prognosis of patients with coronary artery involvement after conservative treatment was poor. Therefore, we recommend carrying out revascularization treatment as soon as possible. The choice of the operation time is generally suitable for the inactive period of TA, and the risk of postoperative vascular restenosis is high in the active status. If the coronary artery stenosis is serious and the lesion is unstable, early surgery should be considered even in the active period to avoid cardiovascular events. However, glucocorticoid and immunosuppressant treatment must be provided at the same time. PCI was safe and effective for our patients, with a high success rate, but the incidence of in-stent restenosis was high. The mortality of the interventional therapy group was 26.3%. CABG can improve angina pectoris and left ventricular ejection function, and the curative effect is stable. Percutaneous transluminal angioplasty or stent implantation has a good short-term curative effect, but it has the possibility of postoperative restenosis, and it can be used as a transitional treatment for CABG. The prognosis of patients with cerebrovascular emergencies was poor in our study. If possible, physicians need to prevent patients from being complicated by cerebral infarction.

In the present study, the subclavian arteries were mostly involved, and the vein bridge was the most commonly used vessel. However, the long-term patency rate of venous bridge vessels is low, and patients with TA are mostly young. In the future, the risk of bridge vessel occlusion will increase yearly. Drug-eluting stents are gradually being used in the treatment of TA involving the coronary artery. Immunosuppressive drugs contained in drug-eluting stents can theoretically inhibit the vascular autoimmune inflammatory response and reduce the occurrence of in-stent restenosis. This study showed that PCI significantly increased the incidence of major cardiovascular events compared with CABG, mainly manifested by restenosis and revascularization. Previous studies have reported that stent implantation can cause chronic static stress and tension, leading to inflammation and cell proliferation, resulting in arterial injury (19–21).

The condition of TA involving the coronary artery and intracranial vessels is complex, with many complications, high risk, and high difficulty in treatment. Treatment options for TA must be carefully selected. If interventional therapy or surgical treatment is selected, angiography must be performed first to clearly understand the exact location, scope, and degree of each arterial stenosis or occlusion. The specific treatment plan according to the actual situation then needs to be determined, and interventional or manual treatment when the ESR is controlled within the normal range should be implemented. If the ESR is not normal, conservative treatment should be used first. The multivariate Cox proportional regression analysis showed that the age of onset, active status, and hypertension were independently associated with composite events. Inflammation plays an important role in the progression and prognosis of TA. In the present study, all the patients were treated with glucocorticoids, and 66 patients (70.2%, 66/94) accepted immunosuppressive therapy. Administration of corticosteroids and other immunosuppressive agents has demonstrated positive anti-inflammatory effects and halting angiographic progression in a majority of patients with TA. Glucocorticoid is the cornerstone of the treatment, especially in the active stage of TA (22–25). It has been reported that immunosuppressive therapy could significantly improve the cardiovascular prognosis of patients with TA (3). Glucocorticoid treatment success rate ranged from 20 to 100% (26, 27). Cyclophosphamide was usually added because of glucocorticoid resistance, serious side effects associated with steroids treatment, or relapse during glucocorticoid dose reduction. Repeated and prolonged courses of immunosuppressive therapy were often required due to the relapsing nature of TA. Antihypertensive agents are frequently used because 52.1% of the patients had hypertension. The active status of TA is often complicated by thrombosis in the affected vessels with stenotic and occlusive lesions. Platelets may be sensitive not only to collagen, but also to prostacyclin because of endothelial dysfunction. Eighty patients (80/94, 85.1%) accept antiplatelet therapy in the present study. Long-term antiplatelet therapy was recommended to prevent thrombus formation in vessels with endothelial damage (28). The prognosis in our study was also affected by cardiac and cerebral diseases. The clinical symptoms, inflammation status, and imaging evaluation should be monitored in TA (29, 30).

## Conclusion

Patients with TA involving both the coronary and intracranial vessels are not rare. This disease can involve any segment of whole blood vessels, but stenosis and occlusion lesions most frequently involve the ostia and proximal segment of the arteries. The prognosis of TA is poor after conservative treatment. Severe vascular lesions should be revascularized as soon as possible. This treatment must be supplemented with glucocorticoids (e.g., prednisone), antiplatelet, nitrates, and statins.

## Data availability statement

The original contributions presented in this study are included in the article/supplementary material, further inquiries can be directed to the corresponding author/s.

## Author contributions

JH, TT, and LY: conceptualization and methodology. JH, BW, LG, and DG: investigation and writing—original draft. JH, RC, JW, and XZ: writing—reviewing and English editing. LG, XZ, and RC: supervision. All authors contributed to the article and approved the submitted version of the manuscript.
